# Supplementary material for: Phylogenetic support of pebS as a phage-exclusive auxiliary metabolic gene
Source: FEMS Microbiol Lett. 2026 Apr 29;373:fnag053. doi: 10.1093/femsle/fnag053 (PMC13198022; doi:10.1093/femsle/fnag053)
Supplement: fnag053_Supplemental_Files [file fnag053_supplemental_files.zip › Supplementary figures_Reviewed.pdf]

Phylogenetic support of *pebS* as a phage-exclusive auxiliary metabolic gene

Nina Baeuerle, Nicole Frankenberg-Dinkel and Anne Kupczok

Supplementary figures

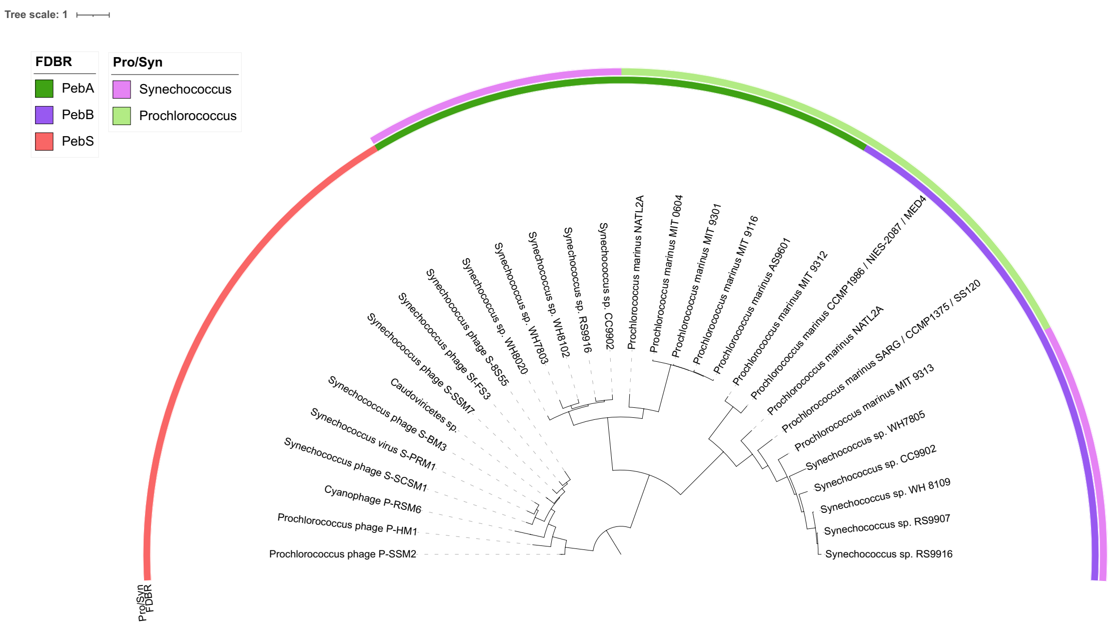

Figure S1: Midpoint rooted maximum likelihood phylogenetic tree with PebS (red), PebB (purple) and PebA (green), test dataset. The tree was constructed using IQ-Tree and visualized with iTOL, midpoint rooted.

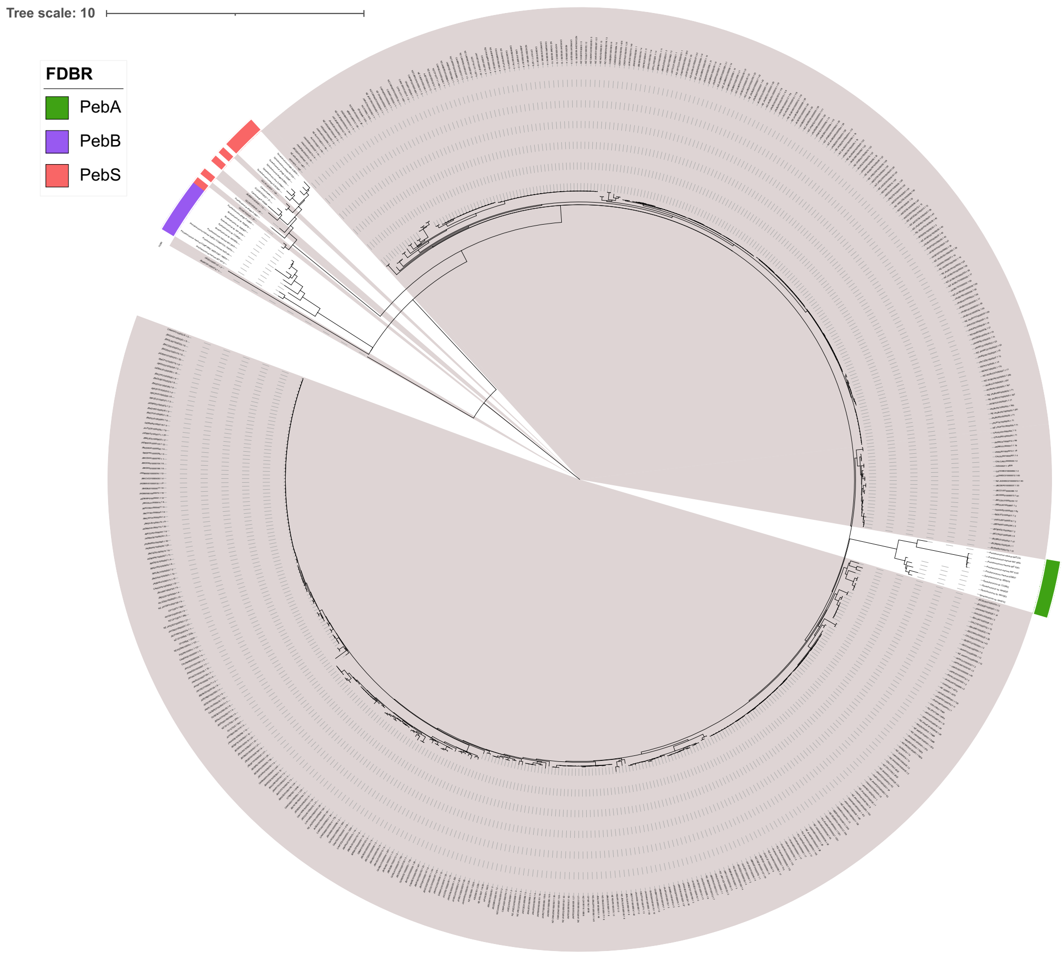

Figure S2: Midpoint rooted maximum likelihood phylogenetic tree with PebS (red), PebB (purple) and PebA (green) data from NCBI sequences (grey background) and a test dataset (marked with the respective colour). The tree was constructed using IQ-Tree and visualized with iTOL.
